# First intention IVF protocol for polycystic ovaries: does oral contraceptive pill pretreatment influence COH outcome?

**DOI:** 10.1186/1477-7827-11-54

**Published:** 2013-06-19

**Authors:** Christine Decanter, Geoffroy Robin, Patricia Thomas, Maryse Leroy, Catherine Lefebvre, Benoit Soudan, Valerie Lefebvre-Khalil, Brigitte Leroy-Martin, Didier Dewailly

**Affiliations:** 1 IVF center of Jeanne de Flandre Hospital, Department of Endocrine Gynaecology and Reproductive Medicine, Centre Hospitalier Régional et Universitaire CHRU, Rue Eugène Avinée, 59037, Lille Cedex, France; 2 Center of Biology and Pathology, Centre Hospitalier Régional et Universitaire CHRU, 59037, Lille Cedex, France; 3 Institute of Reproductive Biology, Centre Hospitalier Régional et Universitaire CHRU, 59037, Lille Cedex, France

**Keywords:** PCO, PCOS, IVF, OCP, Pretreatment, COH outcome, GnRH agonist, Rotterdam ultrasonographic criteria, Oocyte, Embryo

## Abstract

**Background:**

Morphological aspect of polycystic ovaries (PCO) is a very common finding in an IVF center population: this includes PCOS patients identified in 18–25% of the couples presenting with infertility and so called “sonographic PCO only” the prevalence of which has been estimated as high as 33% in asymptomatic patients. Finding the optimal first intention IVF protocol for polycystic ovaries patients is still challenging in order to improve the controlled ovarian hyperstimulation (COH) outcome while avoiding ovarian hyperstimulation syndrome (OHSS). It has been suggested that women with PCO would benefit from a longer period of pituitary down-regulation. The purpose of this study was to compare an extended duration of OCP pretreatment with a classic GnRH agonist protocol.

**Methods:**

A single center prospective non-randomized study was performed from January 2009 to December 2010 in the Lille University Hospital including 113 women diagnosed with PCO(S) according to the Rotterdam ultrasonographic criteria and undergoing their first IVF attempt. Comprehensive hormonal and ultra-sonographic assessments were collected during COH in these patients. LH and androgen suppression and dynamics of follicular growth were compared between the two protocols as well as the COH outcome in terms of oocyte/embryo number and quality, implantation and pregnancy rates.

**Results:**

No significant difference was observed between the two groups concerning dynamics of follicular growth and hormonal values. Clinical and ongoing pregnancy rates were significantly lower in the OCP group despite same oocyte and embryo quality. Nevertheless, the cumulative pregnancy rate did not differ between the two groups. The incidence of OHSS was not statistically significant.

**Conclusions:**

Extended duration of OCP pretreatment, as a first intention IVF protocol for PCO patients, does not improve the pattern of follicular growth nor the oocyte and embryo quality.

## Background

The morphological aspect of polycystic ovaries according to the ultra-sonographic Rotterdam criteria [1] is a very common finding in an IVF center population: this includes PCOS patients identified in 18–25% of the couples presenting with infertility [2] and so called “sonographic PCO only” the prevalence of which has been estimated as high as 33% in asymptomatic patients [3-5]. PCO are characterized by a significantly enlarged cohort of early-growing and recruitable follicles that leads to an increased risk of cycle cancellation and/or hyperstimulation syndrome (OHSS) [6-8]. This excessive follicle number is linked to folliculogenesis disturbances which are presumably the consequence of intra-ovarian hyperandrogenism [9-11]. In addition to the OHSS risk, growth of these follicles during COH is frequently inhomogeneous and the ratio between the number of mature follicles and that of mature oocytes is sometimes disappointing. Consequently, the fertilization rate is often lower than expected [8,9,12].

Finding the optimal first intention IVF protocol for PCO patients, in order to decrease the OHSS risk and to optimize follicular growth and oocyte quality is still challenging and debatable. Few studies indeed have specifically addressed this key point. It has been suggested that women with PCOS would benefit from a longer period of pituitary down-regulation to better suppress LH and androgen levels since the latter have been suggested to be the main culprit for follicle excess [13-15]. Previously, a long duration suppression protocol, which sequentially combined an oral contraceptive pill (OCP) and then daily injections of GnRH agonist, has been designed according to this hypothesis in high responder patients having either PCO or PCOS [16]. By using this protocol, the authors showed improvements in the IVF outcome in terms of implantation and pregnancy rates in patients who previously experienced high responses or OHSS during COH, in comparison to the classic GnRH agonist scheme [16]. Furthermore, the incidence of severe OHSS was significantly reduced. They hypothesized that the longer duration of suppression provided by OCP pre-treatment had improved oocyte quality through better conditions of follicular growth.

The aim of the current study was to prospectively investigate the effects of a longer duration of suppression by an extended OCP pre-treatment versus classic GnRH agonist on the hormonal levels, dynamics of follicular growth and COH outcomes regarding oocyte number and maturity in 113 PCO patients treated for their first IVF attempt.

## Methods

### Study design

This was a single center prospective non-randomized study performed in the IVF center of the Lille University Hospital (France) from January 2008 to December 2009. This study was approved by the institutional review board of Lille University hospital. Written, informed consent was obtained from all subjects before beginning controlled ovarian hyperstimulation (COH).

### Subjects

A total of 113 patients undergoing their first in vitro fertilization (IVF or ICSI) attempt were prospectively recruited according to the Rotterdam ultrasonographic criteria of polycystic ovaries (PCO) i.e. at least 12 antral follicles (2–9 mm) per ovary and/or ovarian volume equal or higher than 10 cc per ovary [1]. Additional inclusion criteria were: age ≥ 18 and ≤ 37 years old and body mass index (BMI) equal or under 35 kg/m^2^. The indications of IVF or ICSI are detailed in Table 1. Eleven patients were secondarily excluded for various reasons: spontaneous pregnancy, unsuccessful testicular sperm extraction, cervix cancer diagnosis, legal problems. Thus, 102 cycles were usable for statistical analysis. All patients have had D3 baseline hormonal investigations before the attempt, at least 3 months after any hormonal treatment. Results are compiled in Table 1.

**Table 1:** Clinical and baseline hormonal features

	**Non OCP group**	**OCP group**	**p-value**
Number	n = 57	n = 45	
Age (years)	30 [23.9-35.0]	28 [22.0-35.6]	NS
BMI (kg/m^2^)	23 [18.5-33.1]	23 [18.4-32.8]	NS
AFC (n)	38 [25.9-74.3]	38 [26.4-83.0]	NS
Testosterone (ng/mL)	0.37 [0.18-0.81]	0.37 [0.13-0.77]	NS
Δ4 androstenedione (ng/mL)	1.76 [0.89-2.83]	1.84 [0.80-3.01]	NS
FSH (IU/L)	6.2 [3.7-8.9]	5.9 [3.9-8.2]	NS
AMH (pmol/mL)	52.2 [18.2-132.5]	57.2 [14.9-138.9]	NS
PCOS	52.6% (n = 30)	66.7% (n = 30)	NS
IVF/ICSI indications	-male infertility: 66%	-male infertility: 69%	NS
	-tubal infertility: 16%	-tubal infertility:11%	
	-ovulation induction failure: 18%	-ovulation induction failure: 20%	
ICSI	66.7% (n = 38)	68.8% (n = 31)	NS

*NS* Non significant, *BMI* body mass index, *AFC* antral follicle count, *AMH* antimullerian hormone, *PCOS* polycystic ovary syndrome (diagnostic criteria. Rotterdam 2004), *ICSI* intracytoplasmic sperm injection.

Quantitative data presented are medians [5^th^ – 95^th^ percentiles].

No randomization was used to include a patient in a protocol rather than in the other: patients were allocated either to a classic GnRH agonist protocol (“non-OCP group”; n = 57) or to an OCP pretreatment/agonist protocol (“OCP group”; n = 45) according to the prescribing habits of each physician. Four different physicians enrolled the patients for the study. The results were compiled and further analyzed at the end of the inclusion period.

In the whole population, 60 women had PCOS (i.e. PCO and oligoanovulation and/or hyperandrogenism) and 42 had “PCO only” (i.e., ovulatory cycles and no hyperandrogenism).

### Controlled ovarian hyperstimulation protocol

**Figure 1:**
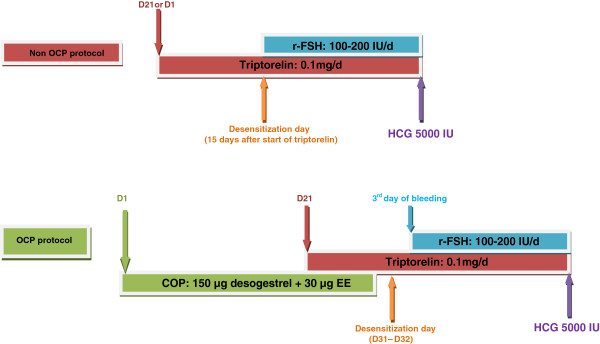
**OCP and non-OCP protocols.** Detailed legend: In the non-OCP group, daily injections of triptorelin 0.1 mg were started in the mid-luteal phase of the preceding cycle (for women with regular menstruations) or on the first day of bleeding (for women with oligomenorrhea or amenorrhea). Desensitization was checked 12 to 15 days after initiation of GnRH agonists (Desensitization day). Daily injections of recombinant FSH (r-FSH) were started only if E2 levels were lower than 50 pg/ml and if there was no functional ovarian cyst. In the OCP group, patients received 28 consecutive days of a monophasic combined oral contraceptive pill (ethinylestradiol (EE) 30 μg and desogestrel 150 μg) starting on cycle day 2. Daily injections of triptorelin 0.1 mg were started on the twenty-first day of OCP. Down-regulation was confirmed 3 or 4 days after discontinuing OCP. Recombinant FSH (r-FSH) was started on the third day of menstrual bleeding. r-FSH starting dose varied between 100 to 200 IU, according to age, BMI and antral follicle count (AFC) in both arms. 5000 IU of purified urinary hCG was administered as soon as at least three follicles reached a mean diameter higher or equal than 17 mm with a consistent rise in serum oestradiol concentration.

The controlled ovarian hyperstimulation protocols are detailed in Figure 1.

In the non-OCP group, daily injections of triptorelin 0.1 mg were started in the mid-luteal phase of the preceding cycle (for women with regular menstruations) or on the first day of bleeding (for women with oligomenorrhea or amenorrhea). Desensitization was checked 12 to 15 days after initiation of GnRH agonists (Desensitization day). Daily injections of recombinant FSH (r-FSH) were started only if E2 levels were lower than 50 pg/ml and if there was no functional ovarian cyst.

In the OCP group, patients received 28 consecutive days of a monophasic combined oral contraceptive pill (ethinylestradiol (EE) 30 μg and desogestrel 150 μg) starting on cycle day 2. Daily injections of triptorelin 0.1 mg were started on the twenty-first day of OCP. Down-regulation was confirmed 3 or 4 days after discontinuing OCP. Recombinant FSH (r-FSH) was started on the third day of menstrual bleeding.

r-FSH starting dose varied between 100 to 200 IU, according to age, BMI and antral follicle count (AFC) in both arms. 5000 IU of purified urinary hCG was administered as soon as at least three follicles reached a mean diameter higher or equal than 17 mm with a consistent rise in serum oestradiol concentration.

Oocyte retrieval was performed 35–36 h after hCG injection by transvaginal ultrasound-guided needle aspiration. Concerning IVF procedure, ICSI was performed in cases with severe male infertility. All embryo transfers were performed 2 or 3 days post-oocyte retrieval. “Top quality” embryos required all the following criteria: presence of 4–5 blastomeres on day 2 and at least 7 on day 3, blastomeres of equal size, absence of multinucleated blastomeres and less than 10% anucleated fragments [17].

Luteal phase support consisted in 600 mg vaginal micronized progesterone per day and 6 mg oral oestradiol per day, both initiated from the retrieval day. If a pregnancy occurred, progesterone and estrogen administration was maintained up to the evidence of fetal heart activity at ultrasound. Clinical pregnancy rate was defined as a pregnancy confirmed by the presence of a gestational sac with an embryo by ultrasound. Implantation rate is defined by the ratio between the number of gestational sacs and the number of embryos transferred. Ongoing pregnancy rate is defined as a pregnancy that has progressed beyond the critical first trimester. Clinical and ongoing pregnancy rates were defined per oocyte retrieval. The cumulative clinical and ongoing pregnancy rates represented the number of clinical and ongoing pregnancies resulting from the fresh and frozen-thawed embryo transfers per oocyte retrieval.

OHSS cases were classified as mild, moderate and severe. Patients were considered to have a moderate or severe ovarian hyperstimulation syndrome (OHSS) if they had suggestive clinical, ultrasound and/or biological signs requiring at least a regular monitoring and initiation of specific treatment.

All the patients underwent a minimal ovarian stimulation for the frozen-thawed embryo transfer. They started 50 IU of r-FSH on day 2 of menstrual bleeding (after 10 days of natural progesterone if they were amenorrheic). An ultrasonographic exam was performed at day 11 or 12. HCG triggering was decided if there was a follicle of at least 17–18 mm of diameter. Day 2/day 3 embryos transfer was done 5 days after HCG.

### Hormonal assays and ultrasound examination

Serum samples were collected and frozen for subsequent assays of LH, estradiol (E2), progesterone, testosterone and Δ4-androstenedione on desensitization day, day 7 and the day of HCG (HCG-D). LH was measured using chemiluminescent, two-site immunoassays on a multiparameter system (Axsym; Abbott Laboratories, Rungis, France). E2, progesterone, testosterone and Δ4-androstenedione were measured by using immunoassays as described previously [18,19].

During each monitoring, ultrasound measurements were performed according to a standardized protocol. Follicles were counted and classified in 5 different size categories: less than 6 mm; 6–9 mm; 10–12 mm; 13–15 mm and higher than 15 mm using the same ultrasound scan (Philips IU 22, France).

### Statistical analysis

Continuous variables were expressed as medians and 5^th^–95^th^ percentiles. Because of non-Gaussian distribution, they were compared with the non-parametric Mann-Whitney test. Categorical variables and frequencies were compared with the chi-square test. A p value of <0.05 was considered significant.

## Results

### Ultrasonographic and hormonal data

Clinical and hormonal characteristics of all patients are shown in Table 1. No significant difference was observed between non-OCP group and OCP group regarding age, BMI, AFC, basal follicle stimulating hormone (FSH), anti-mullerian hormone (AMH) and androgen levels and primary indication of IVF. The prevalence of PCOS was not significantly different between the 2 groups (52% vs 66%, respectively).

**Figure 2:**
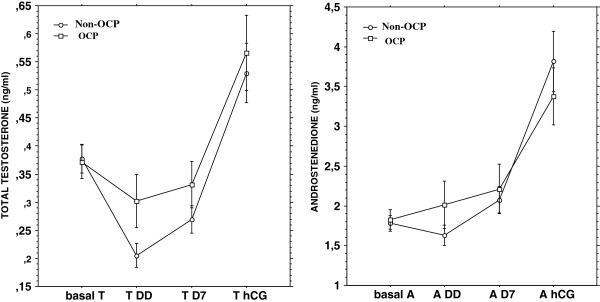
**Dynamic variations of serum total testosterone and delta4androstenedione throughout COH.** Detailed legend: there was no statistical difference between the 2 groups regarding serum delta 4 androstenedione and total testosterone levels throughout.

Follicular growth dynamics and hormonal profile during COH are shown in Table 2. Distribution of different follicular size categories on desentization day (DD), day 7 (D7) and HCG-D was similar in both groups. E2, androgens and progesterone serum levels on DD, D7 and HCG-D during COH, did not differ significantly between non-OCP and OCP groups. The androgen variations throughout COH are represented in Figure 2. Total testosterone levels were higher in the OCP group presumably because of the hepatic effects on SHBG. But the difference between the 2 groups was not statistically significant. Median serum LH level at DD was significantly lower in the OCP group than in the non-OCP group (Table 2). It was similar in the two groups on D7 and HCG-D.

**Table 2:** Follicular growth and hormonal profile during COH

		**Non OCP group**	**OCP group**	**p-value**
**Desensitization day**	<6 mm follicles	29 [15.2-54.3]	26 [15.7-65.8]	NS
	6–9 mm follicles	5 [1.0-14.4]	6.5 [2.0-18.6]	NS
	**LH (IU/L)**	**1.7 [0.6-4.3]**	**0.7 [0.5-2.5]**	**P = 0.0001**
	Prog (ng/mL)	0.2 [0.1-0.6]	0.3 [0.1-0.6]	NS
	Testo (ng/mL)	0.19 [0.05-0.45]	0.23 [0.06-1.06]	NS
	Δ4 (ng/mL)	1.71 [0.86-3.13]	1.48 [0.74-5.40]	NS
**D7**	6–9 mm follicles	11 [2.1-30.1]	10 [3.9-40.5]	NS
	≥10 mm follicles	6 [1.1-17.0]	6 [1.0-23.0]	NS
	E2 (pg/mL)	396 [76.4-1269]	287 [94.7-1680.9]	NS
	LH (IU/L)	0.9 [0.5-2.4]	0.7 [0.3-1.1]	NS
	Prog (ng/mL)	0.3 [0.1-0.6]	0.3 [0.2-0.5]	NS
	Testo (ng/mL)	0.27 [0.10-0.49]	0.32 [0.05-0.55]	NS
	Δ4 (ng/mL)	2.01 [0.87-4.03]	2.05 [0.82-4.17]	NS
**HCG-D**	10–12 mm follicles	7 [3.0-13.9]	4 [1.0-15.0]	NS
	13–15 mm follicles	7 [2.0-23.8]	7 [2.0-18.2]	NS
	>15 mm follicles	8 [4.5-15.5]	9 [3.0-15.0]	NS
	E2 (pg/mL)	3135 [1704.0-5528.1]	3131 [1120.5-5859.0]	NS
	LH (IU/L)	1.4 [0.5-2.2]	1.0 [0.4-2.3]	NS
	Prog (ng/mL)	0.8 [0.3-2.3]	0.7 [0.2-1.4]	NS
	Testo (ng/mL)	0.46 [0.31-1.34]	0.53 [0.27-1.39]	NS
	Δ4 (ng/mL)	3.50 [1.78-7.81]	3.69 [1.02-6.72]	NS

*COH* controlled ovarian hyperstimulation, *D7* seventh day of controlled ovarian hyperstimulation, *HCG-D* HCG injection day, *LH* luteinizing hormone, *Prog* progesterone, *Testo* testosterone, *Δ4* delta-4 androstenedione, *E2* oestradiol. Quantitative data presented are medians [5^th^ – 95^th^ percentiles].

Endometrial thickness on day 1 of r-FSH treatment was significantly lower in the non-OCP protocol and then became significantly higher on HCG day (Table 3). Thus, the degree of endometrial growth (delta between HCG-D and D1) was significantly more pronounced in non OCP protocol (Table 3).

**Table 3:** COH outcome

		**Non OCP group**	**OCP group**	**p-value**
**Clinical outcome**	Cycles (n)	57	45	
	% cancellation before retrieval	15.8% (n = 9)	13.3% (n = 6)	NS
	% oocytes retrieval (n)	84.2% (n = 48)	86.7% (n = 39)	NS
	r-FSH starting dose (IU)	125 [100–200]	125 [100–150]	NS
	Duration of stimulation (days)	11 [8.45-15.55]	11 [9.0-14.0]	NS
	Total dose of r-FSH (IU)	1437.5 [872.5-2555]	1462.5 [1000–2250]	NS
	% OHSS	16.6% (n = 8)	10.2% (n = 4)	NS
	**Days of GnRH agonist (n)**	**25 [20–33]**	**24 [21–29]**	**P = 0.011**
**Endometrium thickness**	**D1 (mm)**	**2.0 [2.0-5.8]**	**3.0 [2.0-6.3]**	**P = 0.001**
	**HCG-D (mm)**	**11.0 [8.5-14.8]**	**10.0 [7.9-13.0]**	**P = 0.002**
	**Delta (mm) (HCG-D – D1)**	**8.0 [4.4-12]**	**6.5 [2.3-10.3]**	**P < 0.0001**
**Oocytes**	Total oocytes (n)	14 [7.5-24.0]	14 [7.0-27.0]	NS
	Lysed oocytes (n)	1 [0–5.6]	2 [0–6.0]	NS
	Meta II oocytes (n)	11 [4.0-19.0]	8 [5.0-22.0]	NS
	Ratio meta II oocytes/≥ 15 mm follicles	1.44 (+/−0.92)	1.32 (+/−0.80)	NS
**Embryos**	Embryos (n)	6 [0.5-14.5]	5 [0.0-13.0]	NS
	Fertilization rate	63% (+/−21)	56% (+/−26)	NS
	“Top quality” embryos (n)	2 [0.0-7.0]	1 [0.0-8.0]	NS
	Cycles with embryo transfer (n)	46	33	
	One embryo transferred (%)	6.5 (n = 3)	18.2 (n = 6)	NS
	Two embryos transferred (%)	87 (n = 42)	81.8 (n = 27)	NS
	Three embryos transferred (%)	2.2 (n = 1)	0 (n = 0)	NS
	Cycles with freezing (%)	58.3 (n = 28)	51.2 (n = 20)	NS
	Frozen embryos (n)	4 [1.0-11.0]	3.5 [1.0-10.0]	NS
	Recovery rate after thawing	72,8%	77,9%	NS
	Frozen-thawed embryo transfers (n)	19	21	
	Clinical pregnancy rate per frozen-thawed embryo transfer	31,6% (n = 6)	38% (n = 8)	NS
	Ongoing pregnancy rate per frozen-thawed embryo transfer	31,6% (n = 6)	38% (n = 8)	NS

*COH* controlled ovarian hyperstimulation, *r-FSH* recombinant FSH, *OHSS* Ovarian hyperstimulation syndrome, *D1* first day of controlled ovarian hyperstimulation, *HCG-D* HCG injection day. meta II: metaphase II. Continuous variables are expressed as medians [5^th^ – 95^th^ percentiles].

All these results did not differ between the two protocols when the study population was divided into 2 groups, i.e. PCOS and PCO-only.

### COH outcome

COH outcome is summarized in Table 3. Cancellation rate was similar between the two protocols. There were 9 cancellations in the non OCP group, 3 for hyper-response, 3 for insufficient response and 3 for desensitization failure. There were 6 cancellations in the OCP group: 4 for hyper-response and 2 for insufficient response. Then after, there was no transfer in 2 cases in the non-OCP group (no embryo in classical IVF for spermatic reasons) and 6 in the OCP group for various reasons: 1 case of OHSS, no or very poor quality embryo. There was no difference between the two groups regarding the duration of stimulation and the total dose of r-FSH

**Figure 3:**
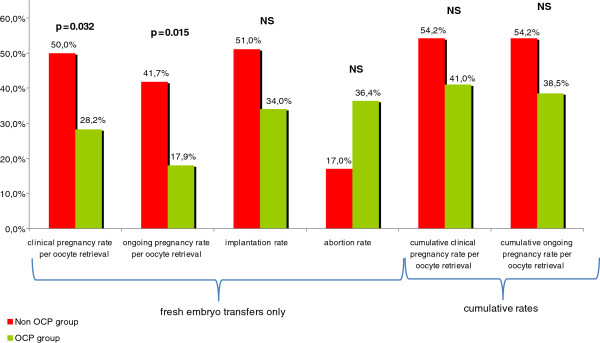
**Comparison of COH outcome in OCP and non-OCP groups.** Detailed legend: Clinical and ongoing pregnancy rates after fresh embryo transfer were significantly higher in the non-OCP protocol (50% *vs* 28.2%, p = 0.032 and 41.7% *vs* 17.9%, p = 0.015, respectively). The implantation rate was higher in non-OCP group but the difference was not statistically significant (51% *vs* 34%). Similarly, the abortion rate was higher in OCP group protocol but not significantly (36.4% *vs* 17%). Cumulative clinical and ongoing pregnancy rates (including frozen embryo transfers) were not statistically different between the two groups (54.2% *vs* 41.0% and 54.2% *vs* 38.5%, respectively).

There was no significant difference concerning the occurrence of OHSS between these two protocols. Duration of GnRH agonist treatment was significantly higher in the non-OCP group than in OCP group (Table 3). Mean free-pill interval in the OCP group was 5 days. After oocyte retrieval, there was no difference in the number of total oocytes, metaphase II oocytes, total embryos, “top quality” embryos between the two groups. The rate of embryo freezing was similar in both groups. Clinical and ongoing pregnancy rates after fresh embryo transfer were significantly higher in the non-OCP protocol (50.0% *vs* 28.2%, p = 0.032 and 41.7% *vs* 17.9%, p = 0.015, respectively) (Figure 3). The implantation rate was higher in non-OCP group but the difference was not statistically significant (51% *vs* 34%). Similarly, the abortion rate was higher in OCP group protocol but not significantly (36.4% *vs* 17%). Live birth rate was per oocyte retrieval after fresh embryo transfer was statistically significant between the 2 groups: 41, 7% vs 17, 9% (p = 0,015). Cumulative live birth rates per oocyte retrieval was not different between the 2 groups: 52, 0% vs 38,5% (p = NS).

Cumulative clinical and ongoing pregnancy rates (including frozen embryo transfers) were not statistically different between the two groups (54.2% *vs* 41.0% and 54.2% *vs* 38.5%, respectively). All these results did not differ between the two protocols when the study population was divided into 2 groups, i.e. PCOS and PCO-only.

## Discussion

### Hormonal and ultrasonographic data

The present study shows that a longer duration of suppression, by using sequentially OCP and GnRH agonist, affects neither the dynamics of follicular growth nor the androgen levels during the first COH in PCO patients, despite significantly lower LH levels on desensitization test day. It has to be noted that LH levels became similar between both groups from the seventh day of COH, suggesting that lower LH levels in the OCP group at desentization day was more related to the association of OCP and GnRh agonist rather than the duration of suppression. Our results are in keeping with those of Damario et al. [16] and Hwang et al. [14] who both proceeded through a longer duration of suppression in high responder and/or PCOS patients. Damario et al. [16] retrospectively investigated dual suppression protocol in 38 patients who had previously experienced high response and/or OHSS with a classic GnRH agonist protocol. Every woman was compared to herself. The authors did not find any hormonal difference regarding testosterone and androstenedione levels despite significantly lower LH levels in the dual suppression group at desensitization day. In the randomized control trial (RCT) of Hwang et al. [14], including 49 PCOS patients undergoing their first IVF attempt, the classic long agonist protocol was compared to a protocol with a longer duration of suppression by combining 3 months of OCP followed by antagonist. Serum androgen levels were suppressed equally in both protocols at the start of gonadotropin administration. In our study, as well as in others [14,16], androgen levels decreased significantly from baseline to desensitization day values, but to a similar extent in both protocols. Thus, a deeper and longer duration of pituitary suppression by using OCP is not able to further suppress the androgen levels. Not surprisingly, and accordingly to the hormonal data, the growth of follicles showed a similar pattern in both groups. We only noted a tendency toward a higher number of intermediary follicles, i.e. 10–12 mm of diameter, in the non-OCP protocol. This could explain the slightly higher incidence of severe OHSS observed in this group. There was significantly less OHSS in the study of Damario et al. [16] but dual suppression was used as a second intention protocol in those patients who previously experienced a hyper response and for whom further r-FSH starting dose adjustments were done.

### COH outcome

No difference was noted between the 2 protocols regarding oocyte number and quality, fertilization rates and embryo number and quality. Nevertheless, pregnancy rates differed drastically with a significantly higher rate in the non-OCP group. It has to be noted that there were slightly more cancellations of embryo transfer in the OCP group because of poor quality embryo. Although the number of poor quality embryos was not significant between the 2 groups, it could have influenced the results. The non-randomized design of this study and the size of the study population make the results difficult to extrapolate but this very significant difference must draw attention. Interestingly, there was no longer any statistical difference when the cumulative pregnancy rate was considered, raising the issue of defective implantation with OCP.

OCP pre-treatment in IVF has been widely used in agonist and antagonist protocols for avoiding ovarian cysts formation, LH surge and for cycle scheduling purposes. There is still some debate regarding the effects of OCP upon the likelihood of pregnancy. To date, only few RCT have been performed to address this issue, all in antagonist cycles. A meta-analysis including 4 RCT did not find any statistical difference regarding pregnancy rates between protocols with or without OCP [20]. Conversely, in a RCT including 425 patients, Kolibianakis et al. [21] observed a significantly higher early pregnancy loss in the OCP pretreatment arm, whereas ongoing pregnancy rates were similar between the 2 groups. Recently, a review from the Cochrane database suggested that combined OCP in GnRH antagonist cycles, compared to no pre-treatment, is associated with fewer clinical pregnancies and poorer pregnancy outcome [22].

Whether OCP could influence the implantation process by direct adverse effects on the endometrium and/or by overly lowering LH levels remains to be elucidated. In the current study, there was no difference concerning LH levels on D7 and HCG-D of COH between the 2 groups. Conversely, as reported by Kolibianakis et al. [21], we observed significant differences between OCP and non-OCP groups concerning both the kinetic of growth and the thickness of the endometrium during COH. The mean delta between the endometrial thickness on HCG day and on DD was significantly different between the 2 groups. In OCP group, in comparison with the other group, the endometrium was thicker on DD, but thinner on HCG day. This difference could be attributed either to a direct adverse effect of OCP on the endometrium and/or to the menstrual pattern after pill discontinuation. In the current study the mean free-pill interval was 5 days, but it has to be mentioned that about 5% of patients didn’t bleed at all after pill discontinuation.

Damario et al. [16] showed improvements in implantation and pregnancy rates by using OCP pre-treatment which is different from our results. This could be explained by differences regarding the design of the study and by the fact they used fewer days of OCP.

The prevalence of PCOS was slightly higher in the OCP group (66% vs 52%) but the difference was not statistically significant. Thus, the hypothesis that it could have interfered with our results is unlikely. Moreover, even though data from head to head comparisons between PCOS and PCO-only during COH are scarce, studies that have addressed this issue showed similar numbers of oocytes, the same implantation and pregnancy rates and the same OHSS risk [3,23-25]. Lastly, when we divided the study population into 2 groups, i.e. PCO-only and PCOS, differences between the two protocols remained the same compared with the whole population. This confirms that therapeutic management of sonographic PCO for IVF in terms of protocol and r-FSH starting dose choices may be the same as for PCOS.

Very few studies have been conducted in specific subgroups of PCO and/or PCOS patients, whereas its prevalence in an IVF center population is high. In the current study, we chose to consider only the first IVF attempts because we think it is definitely challenging and of high importance to find the optimal, i.e. safe and effective, first intention IVF protocol in this context.

## Conclusion

In conclusion, extended OCP pretreatment, as a first intention IVF protocol for PCO patients, does not improve the pattern of follicular growth nor the oocyte and embryo quality. Whether OCP pretreatment could be detrimental on the COH outcome regarding likelihood of pregnancy through endometrial adverse effects remains to be confirmed through a randomized control study in a much larger and well-powered population.

## Abbreviations

COH: Controlled ovarian hyperstimulation; IVF: In vitro fertilization; PCO: Polycystic ovaries; PCOS: Polycystic ovary syndrome; OCP: Oral contraceptive pill; OHSS: Ovarian hyperstimulation syndrome.

## Competing interests

The authors declare that they have no competing interests.

## Authors’ contributions

CD conceived of the study, participated in its design and coordination. She drafted the manuscript. GR worked on the collection of information and helped to achieve the statistical analysis. PT, ML, and CL participated in patient’s recruitment. PP and BS performed all hormonal measurements. DD performed the statistical analysis. All authors read and approved the final manuscript.

## Authors’ information

CD is a medical doctor in endocrinology and reproductive medicine. She practices in the IVF center of the Lille University Hospital, “Hôpital Jeanne de Flandre,”France. She is the head of the fertility preservation unit. Her special interests are in IVF protocols in PCO, hormonal markers during COH and fertility preservation. GR, ML, PT and CL are practitioners of the Lille University hospital IVF center. PP and BS are practitioners in the biology pathology center. DD is the head of reproductive medicine unit at the Lille University Hospital. He is endocrinologist. His main special interest concerns PCO.
